# Tactile-Sensation Imaging System for Assessing Material Inclusions in Breast Tumor Detection

**DOI:** 10.3390/bios16020102

**Published:** 2026-02-04

**Authors:** Tahsin Nairuz, Jong-Ha Lee

**Affiliations:** Department of Biomedical Engineering, Keimyung University, Daegu 42601, Republic of Korea; tahsinnairuzdu11@gmail.com

**Keywords:** breast tumor, inclusion analysis, polydimethylsiloxane optical waveguide, tactile-sensation imaging system, Young’s modulus

## Abstract

Accurate identification and characterization of subcutaneous tumors are essential for improving breast tumor detection and treatment. This study introduces an innovative Tactile-Sensation Imaging System (TSIS) designed, implemented, and tested to detect and characterize subcutaneous inclusions simulating breast tumors. The system employs a multilayered polydimethylsiloxane (PDMS) optical waveguide that mimics the tactile structure of the human fingertip. By introducing light at a critical angle, the design enables continuous total internal reflection (TIR) within the flexible, transparent waveguide. When external pressure is applied, deformation of the contact area causes light scattering, which is recorded using a high-definition camera and processed as tactile images. Analysis of these images allows estimation of inclusion characteristics such as size, depth, and mechanical properties, including Young’s modulus. Analytical modeling and numerical simulations validated the optical performance of the waveguide, while experimental evaluations using realistic tissue phantoms confirmed the system’s ability to accurately detect and quantify embedded inclusions. The results demonstrated reliable estimations of inclusion dimensions, depths, and stiffness, verifying the system’s sensitivity and precision. The TSIS offers a noninvasive, portable, and cost-efficient solution for quantitative breast tumor assessment, bridging the gap between manual palpation and advanced imaging, with future enhancements aimed at improving resolution and diagnostic accuracy.

## 1. Introduction

Breast cancer remains one of the most prevalent and serious health challenges worldwide, marked by its escalating incidence and substantial mortality burden. According to the World Health Organization, an estimated 2.3 million new cases of breast cancer were diagnosed globally in 2022, resulting in around 670,000 deaths [1]. Moreover, the International Agency for Research on Cancer (IARC) projects that if current trends continue, global breast cancer incidence will increase by 38% and associated deaths by 68% by 2050 [2], highlighting a critical escalation in the global disease burden. The disease is most common among women aged 40–55 and remains the second leading cause of cancer death among women in general [3]. The major histopathological classifications encompass ductal carcinoma in situ, lobular carcinoma in situ, infiltrating ductal carcinoma, and infiltrating lobular carcinoma, whereas mucinous, medullary, and tubular carcinomas are comparatively uncommon [4]. Inflammatory breast cancer and Paget’s disease of the nipple are exceedingly rare, comprising roughly 1% of total diagnoses, while phyllodes tumors occur even less frequently, accounting for fewer than 10 deaths annually in the United States [5,6]. The rising global incidence accentuates the imperative for early detection and equitable access to advanced diagnostic modalities to mitigate the disease burden and improve survival outcomes worldwide.

At present, several methods are employed for breast cancer screening, and extensive research endeavors continue to refine detection technologies according to criteria emphasizing diagnostic accuracy, high sensitivity, acceptable specificity, procedural simplicity, patient comfort, temporal efficiency, and cost-effectiveness [7]. The traditional practice of breast self-examination (BSE) remains a widely advocated approach for preliminary tumor identification. In comparison, clinical breast examinations (CBEs) performed by trained healthcare professionals exhibit diagnostic success rates exceeding 57%, alongside specificity surpassing 97% [8,9], thus demonstrating considerable clinical reliability. Although these screening modalities cannot independently determine the malignancy of a lesion, they play a pivotal role in detecting abnormalities necessitating further diagnostic evaluation. Nonetheless, a fundamental limitation of CBE lies in the clinician’s inherent difficulty in articulating and standardizing tactile observations, thereby constraining the objectivity and reproducibility of the examination outcomes [8].

In modern clinical practice, noninvasive imaging modalities—including mammography, ultrasound, magnetic resonance imaging (MRI), and elastography—are routinely used for breast cancer detection and diagnostic evaluation. Mammography, which has remained the gold standard for more than three decades, employs controlled breast compression and low-dose X-rays to achieve high true-positive detection rates [10]; however, its diagnostic accuracy is markedly influenced by tissue density, body mass index (BMI), age, and genetic predisposition [11,12,13]. Ultrasound imaging, widely adopted for evaluating palpable breast masses and characterizing cystic versus solid lesions, is particularly valuable in pregnant and lactating women because it avoids ionizing radiation; nonetheless, its diagnostic performance is constrained by operator dependency and reduced image clarity due to speckle noise and poor lesion margin delineation [14,15]. MRI offers superior two- and three-dimensional visualization and excellent soft-tissue contrast without radiation exposure, yet it remains cost-intensive and often necessitates contrast enhancement and expert interpretation [16,17]. In parallel, elastography has emerged as a promising adjunct technique, enabling quantitative assessment of tissue stiffness and thereby enhancing the specificity and diagnostic accuracy of ultrasound and MRI when used in combination [18].

Building upon the aforementioned advancements in imaging technologies, the breast nodule stiffness has emerged as a critical diagnostic biomarker for breast cancer, as elevated tissue rigidity is strongly correlated with increased malignancy potential [19]. Conventional diagnostic approaches—most notably CBE via manual palpation—remain integral to preliminary screening, where clinicians rely on tactile cues to record findings through qualitative descriptions or schematic illustrations. However, such methods are inherently subjective, as they depend heavily on the examiner’s tactile sensitivity and interpretive experience, thereby limiting reproducibility, quantification, and inter-examiner consistency [20].

To address these diagnostic limitations, the present study introduces the Tactile Sensation Imaging System (TSIS)—a novel, noninvasive optical-based screening platform designed to detect and characterize subcutaneous inclusions associated with breast tumors. This system quantitatively determines critical tumor parameters—including size, depth, and Young’s modulus—by leveraging tactile sensation imaging to generate objective, high-resolution representations of tissue stiffness. TSIS translates the tactile feedback principle of CBE into a digital imaging framework enhanced by optical waveguide-based sensing and advanced data processing algorithms, thereby offering quantifiable and reproducible assessments of tumor geometry and mechanical properties.

Beyond its diagnostic precision, TSIS distinguishes itself through its cost-effectiveness, portability, and operational simplicity, positioning it as a viable tool for low-resource clinical environments. Unlike conventional imaging systems such as mammography, ultrasound, elastography, or MRI—which depend on costly X-ray tubes, high-frequency transducers, or superconducting magnets—TSIS operates using a compact optical setup and does not require ionizing radiation or contrast agents. This reduces both equipment and maintenance costs while minimizing procedural risks. TSIS offers an economically sustainable, radiation-free, and technologically scalable alternative for early breast tumor assessment, with performance characteristics that support its potential as a complementary screening approach [21]. The following sections describe the design concept of TSIS, detailing its operational principles, imaging mechanism, and numerical simulations that substantiate the theoretical foundation of the system. Furthermore, the system’s effectiveness is experimentally validated using tissue-mimicking phantoms containing inclusions of different sizes, depths, and elastic moduli to verify the efficacy of the proposed approach, demonstrating its ability to replicate real tissue conditions for improved diagnostic performance.

## 2. Research Subjects and Methods

### 2.1. System Design

The TSIS comprises four main components: an optical waveguide, a light-emitting source, a high-resolution imaging camera, and an advanced processing unit. An optical waveguide operates as the core-detection element of the system. Constructed from polydimethylsiloxane (PDMS), an excellent silicone material, the waveguide is flexible and transparent, which are the key characteristics that meet the requirements for tactile sensation imaging.

To approximate the tactile sensitivity of humans, the system design mimics the structure of the human fingertip, comprising three layers with varying elastic properties: the outermost epidermis, intermediate dermis, and innermost subcutaneous tissue. The epidermis, although the most rigid layer, exhibits a relatively low elastic modulus and is approximately 1 mm thick. Beneath this layer is the dermis, which is more flexible and ranges in thickness from 1–3 mm. The subcutaneous tissue, which is the softest layer, serves as a cushion and is primarily composed of fat. This layered structure allows the inner tissues to deform more than the outer layers when pressure is applied. To replicate this structure, the TSIS incorporated three PDMS layers with varying hardness values. The first PDMS layer was the hardest, the second was of intermediate hardness, and the third was the softest, with thicknesses of 2, 3, and 5 mm, respectively.

**Figure 1:**
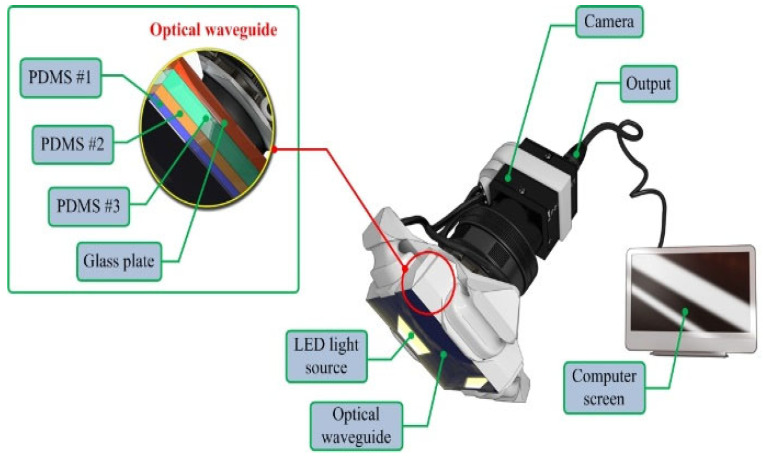
Tactile-sensation imaging system. The system consists of a multilayer PDMS optical waveguide (PDMS #1–#3; thicknesses of 2, 3, and 5 mm) bonded to a borosilicate glass substrate and illuminated by four peripheral micro-LEDs (3 mm diameter) to establish total internal reflection (TIR). Local surface deformation perturbs the TIR condition, generating deformation-dependent light scattering. A high-resolution monochrome CMOS camera (1392 × 1042 pixels, 60° field of view) positioned beneath the waveguide and protected by the glass substrate captures the scattered light patterns. The acquired images are transmitted to a computer for real-time visualization and quantitative processing.

The high-definition camera employed in TSIS is a monochrome-cooled complementary metal-oxide-semiconductor (CMOS) sensor featuring a pixel configuration measuring 4.65 μm × 4.65 μm. The camera provides a maximum resolution of 1392 (H) × 1042 (V) pixels at a 60° viewing angle. Positioned beneath the waveguide, the camera is protected by a thermally resistant borosilicate glass panel, which ensures that the properties of the waveguide do not degrade the camera resolution. Illumination was provided by four micro-LED light sources, each with a 3 mm diameter, positioned on the periphery of the waveguide. These light sources were calibrated to achieve optimal angles of incidence for total internal reflection (TIR) within the waveguide. A comprehensive schematic of the TSIS design is presented in Figure 1, and the key system components and specifications are summarized in Table 1 to highlight the originality and reproducibility of the proposed system.

Prior to experiments, the TSIS was calibrated to ensure stable optical and mechanical measurements. Optical calibration was performed by adjusting the peripheral micro-LEDs to achieve incidence angles satisfying total TIR within the multilayer PDMS waveguide. Spatial calibration was conducted using a reference target to establish the pixel-to-distance conversion factor for quantitative image analysis. Mechanical calibration was performed under quasi-static loading conditions by applying controlled forces to the waveguide surface and correlating the resulting deformation-induced pixel intensity with applied force. These calibration steps were conducted under controlled lighting and temperature conditions to minimize environmental variability.

### 2.2. Operating Principle of the Imaging Mechanism

The TSIS is based on the extensively validated and well-established concept of TIR. According to Snell’s law, when light travels through a boundary between two materials with different refractive indices, light is partly reflected, and partly transmitted. When the angle of incidence exceeds the critical angle, TIR occurs, resulting in the light being fully reflected within the medium.

**Figure 2:**
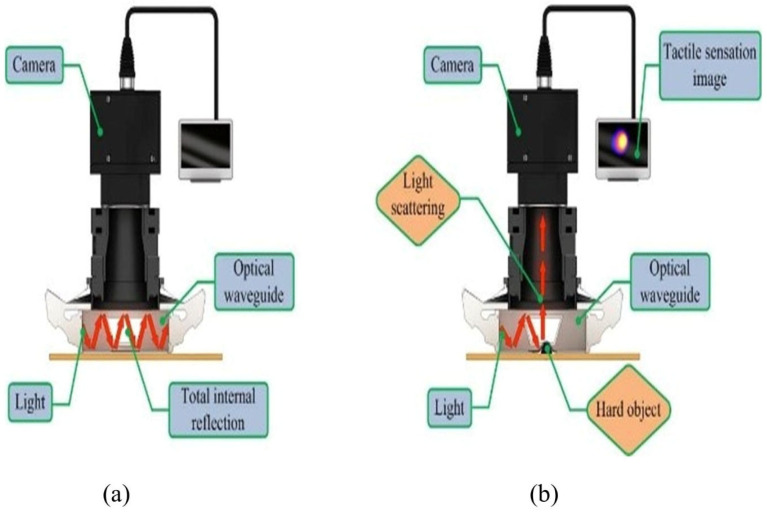
Imaging principle of TSIS. (**a**) Propagation of light within the waveguide owing to total internal reflection. (**b**) Deformation of the waveguide due to an external force resulting in light scattering.

In the TSIS, the PDMS waveguide is encased in air and has a lower refractive index than the waveguide material. This allows TIR to occur as light propagates through the waveguide. When the waveguide comes into contact with a solid object, it becomes deformed; this results in scattering of the light. The high-definition camera subsequently records the scattered light, converting the changes into an image (Figure 2). The primary imaging mechanism relies on detecting scattered light caused by variations in the critical angle due to external pressure.

### 2.3. Optical Analysis of the Imaging Principle

To gain further insight into this imaging technique, we conducted an optical evaluation. The optical characteristics of a single-layer waveguide were examined initially; then, the model was extended to a four-layer waveguide. Below, we present the numerical simulations for validating the model.

#### Numerical Simulation

The next step in validating the imaging principle was to perform numerical simulations based on the analytical model described in Supplementary Files. We numerically demonstrated the propagation of light under TIR conditions within the multilayer waveguide. The impact of waveguide deformation on light scattering was also simulated.

**Figure 3:**
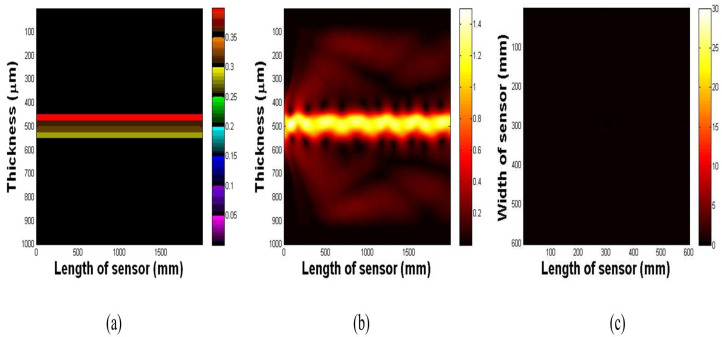
(**a**) Side view of the optical waveguide. (**b**) Light oscillating inside the waveguide as predicted by Snell’s law. (**c**) Top surface image showing the absence of scattering.

Figure 3a illustrates the waveguide prior to light injection, highlighting the distinct PDMS and glass layers. As depicted in Figure 3b, upon the injection of light, a portion of the light undergoes diffraction owing to discontinuities at the air-waveguide interface. However, most of the light undergoes TIR, oscillating sinusoidally within the waveguide. As shown in Figure 3c, top-surface imaging of the waveguide reveals no scattering because the light is fully confined within the structure.

**Figure 4:**
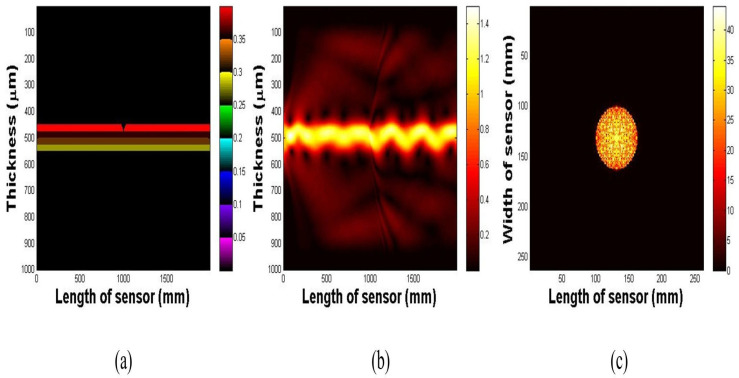
(**a**) Side view of the deformed waveguide. (**b**) Light scattering resulting from the deformation. (**c**) Light scattering from the waveguide surface.

In a subsequent simulation, we introduced a deformation in the waveguide surface, 5 mm in depth and 10 mm in radius. This alteration, as illustrated in Figure 4a, disrupts the light path and causes scattering in the region of the deformation (Figure 4b). The captured image presented in Figure 4c clearly displays the scattered light emanating from the deformed region of the waveguide surface.

### 2.4. Approximation of Geometric Optics

To ensure that TIR occurs within the optical waveguide, we calculated both the critical and acceptance angles using the principles of geometric optics. This analysis assumes that light behaves as a ray, which allows for a more straightforward calculation of the light propagation through the different layers of the waveguide.

**Figure 5:**
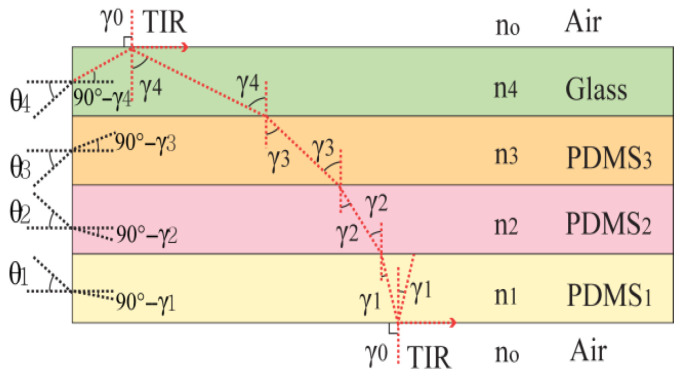
Light propagation as a ray within the waveguide, traveling at angles $\gamma_{i}$ in each layer *i, i* = 0, 1, 2, 3, 4, 5.

According to Snell’s law, the transmission angle $\gamma_{i}$ within every layer is related to the refractive indices $n_{i}$ of the adjacent layers. Figure 5 provides a graphic representation of light propagation through each layer of the waveguide, with the relationships outlined by the equations below:

(1) $$n_{1}\;sin\;\gamma_{1}=n_{0}\;sin\;\gamma_{0},\;$$

(2) $$n_{2}\;sin\;\gamma_{2}=n_{1}\;sin\;\gamma_{1},$$

(3) $$n_{3}\;sin\;\gamma_{3}=n_{2}\;sin\;\gamma_{2},\;$$

(4) $$n_{4}\;sin\;\gamma_{4}=n_{3}\;sin\;\gamma_{3},$$

(5) $${and\;n}_{5}\;sin\;\gamma_{5}=n_{4}\;sin\;\gamma_{4}.$$

In the case of TIR, light must be fully reflected at the boundary between the waveguide and air. For this to happen, the angles of propagation at the boundary, $\gamma_{0}$ and $\gamma_{5}$, must be 90°. This condition results in the lowest possible propagation angle, referred to as the critical angle $\theta_{i}$, for TIR to take place.

The entry angle *θ*_*i*_ represents the largest angle under which light can enter a layer while continuing to be confined within the waveguide. The correlation between the angle of propagation $\gamma_{i}$ and the entry angle $\theta_{i}$ is described by Snell’s law, expressed as follows:

(6) $$sin\;\theta_{i}=n_{i}\;sin\;(90^{{}^{\circ}}-\gamma_{i})\;=n_{i}\;cos\;\gamma_{i},$$

To further refine this equation, we apply the following transformation:

(7) $$sin\;\theta_{i}=n_{i}\;cos\;\gamma_{i}=n_{i}\;{\left(1-\sin^{2}\gamma_{i}\right)}^{½}={\left({n_{i}}^{2}-{{n_{i}}^{2}\sin}^{2}\gamma_{i}\right)}^{½}$$

From Equation (1) through Equation (6), we know that $n_{i}\;sin\;\gamma_{i}$ = $n_{0}\;sin\;90^{{}^{\circ}}$ = 1 (since $sin\;90^{{}^{\circ}}$= 1 for air). Thus, the acceptance angle for each layer *i* can be determined as follows:

(8) $$\theta_{i}=a\;sin\left[{\left({n_{i}}^{2}-1\right)}^{½}\right]$$

Light that enters any layer *i* within this acceptance angle will experience TIR and be confined to the optical waveguide. In the current TSIS design, the indices of refraction for the PDMS layers and the borosilicate glass component were measured as around 1.41, 1.40, 1.39, and 1.38, respectively. Using these values, we calculated the acceptance angles for each layer as follows:

-
$\theta_{1}=83.73{}^{\circ}$

-
$\theta_{2}=78.46{}^{\circ}$

-
$\theta_{3}=74.89{}^{\circ}$

-
$\theta_{4}=71.98{}^{\circ}$

Consequently, ensuring TIR within the waveguide requires that the angle of the spatial radiation pattern of the injected light remains below twice the acceptance angle of the outermost layer, 71.98° × 2 = 143.96°. Therefore, the light sources used in the system were calibrated to ensure that the injected light was within the angular limit.

### 2.5. Tactile Data Processing

In this section, we describe the tactile data processing algorithms that were developed to assess the properties of an inclusion—by analyzing the key attributes derived from the tactile-sensation images. Following the introduction of a sample tactile-sensation image, methods for estimating the comparative size, depth, and Young’s modulus of an embedded inclusion are presented.

#### 2.5.1. Tactile-Sensation Image

**Figure 6:**
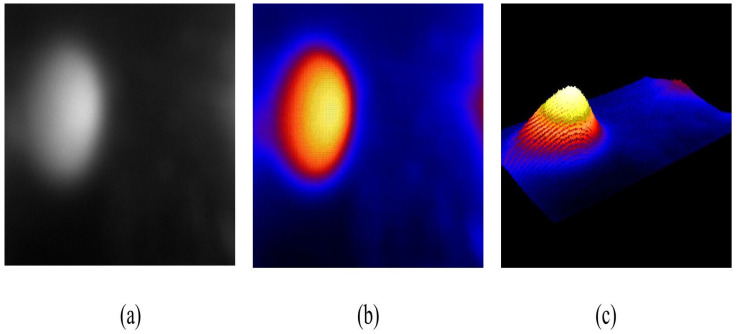
Tactile-sensation imaging for a spherical, soft-polymer inclusion. (**a**) Grayscale tactile image showing deformation-induced light scattering within a 60° field of view; pixel intensity is normaliz and reported in arbitrary units (a.u.). (**b**) Corresponding false-color representation of normalized scattering intensity (a.u.). (**c**) Three-dimensional reconstruction of the tactile response, where the x–y axes denote angular position within the 60° field of view and the z-axis represents relative deformation amplitude (a.u.).

A sample tactile-sensation image was captured using TSIS for a spherical inclusion measuring 2 mm in diameter. Figure 6a presents the original grayscale image, whereas Figure 6b displays an image with grayscale values transformed into a false-color scale to improve visual clarity. Finally, Figure 6c shows the 3D reconstruction of the tactile image for a more detailed analysis.

#### 2.5.2. Inclusion Size Estimation

The inclusions were assumed to be spherical; results of clinical tests by Wellman et al., also showed strong alignment with breast tissue modeling based on the spherical assumption [22,23]. Therefore, the inclusion size was estimated by measuring its diameter.

During the system operation, an increase in the inclusion diameter leads to heightened light scattering because of the greater deformation of the optical waveguide. Therefore, the inclusion diameter was estimated by measuring the area of light scattering in the captured images. Let *I*(*x,y*) denote the intensity (value) of each pixel in the image. The total area of light scattering, *A*, can be derived by counting the number of pixels with values exceeding the threshold *k*;

(9) A=number of pixel values, where I(x,y)≥ k. 

In this study, the threshold, *k*, is set to 5. The relative diameter *d* of the inclusion can subsequently be determined using the following equation:

(10) $$d\;=\;2\sqrt{\left(A/\pi \right)}.\;$$

The parameter *d* indicates the diameter measured in pixel distance. To convert the pixel distance to real-world units, we use a scale factor. The scale factor, calibrated for the system, is $6.79\times 10^{-3}\;$mm per pixel. Consequently, the relative inclusion diameter, *d*, is expressed in millimeters (mm).

#### 2.5.3. Inclusion Depth Estimation

As the depth of the inclusion increases, the extent of light scattering diminishes because of the decreased deformation of the optical waveguide. Given the spherical assumption for inclusion, the intensity values in the tactile image typically exhibit a bell-shaped distribution, peaking at the centroid of the image and gradually tapering as the distance from the centroid increases.

We began by calculating the centroid of the image to determine the relative depth of the inclusion. Let (*X*_*c*_, *Y*_*c*_) denote the x- and y-coordinates of the centroid, respectively. The coordinates are calculated using the following formulas:

(11) $$X_{c}=\frac{\iint_{x,y}I\left(x,y\right)xdxdy}{\iint_{x,y}I\left(x,y\right)dxdy},$$

(12) $$and\;X_{c}=\frac{\iint_{x,y}I\left(x,y\right)ydxdy}{\iint_{x,y}I\left(x,y\right)dxdy}.$$

Once the centroid coordinates are determined, the inclusion depth ℎ is assessed using the intensity value at the centroid:

(13) $$h\;=\;I{(X}_{c},Y_{c}).\;$$

As with the estimation of the inclusion size, the same scale factor was utilized to translate the depth from the pixel distance to real-world measurements, and the result is presented in mm.

#### 2.5.4. Inclusion Hardness Estimation

In materials science, the hardness of a material is often expressed using Young’s modulus, *E*, which represents the relationship between stress and strain:

(14) E = stress/strain. 

Stress is determined by the force applied over a specific area. In this study, we estimated force *F* by utilizing the integrated pixel value *M* obtained from the tactile-sensation image. The correlation between the force, *F*, and the integrated pixel value, *M*, was established during the initialization and calibration processes of the system:

(15) $$F=1.0\times 10^{6}\times (0.056+1.478M)$$

(16) $$M=\iint_{x,y}I\left(x,y\right)dxdy$$

As stress is defined as the force distributed over a specific area, the estimated stress *P* can be computed as follows:

(17) P = F/C, 

where *C* represents the surface area of the optical-waveguide bottom.

The next parameter required to estimate Young’s modulus involves deformation. Deformation is defined as the fractional variation in length resulting from applied stress. In this study, strain was measured in terms of the relative shift between a pair of locations on the tactile-sensation image under varying levels of compression.

To calculate strain *T*, two tactile-sensation images were obtained with varying compression forces. Using Equation (10), the relative diameter *d* of the inclusion was measured for both images. The strain was then determined based on the difference between the two relative diameters.

(18) $$T={\;d}_{1}-d_{2}$$

Finally, the estimated stress, *P*, and strain, *T*, are utilized to compute the relative hardness (Young’s modulus) of the inclusion, with the resulting unit for relative hardness being expressed in Pascals (Pa).

## 3. Experimental Results

This section presents the capability of the TSIS for analyzing inclusions embedded within soft tissue. For this purpose, three uniquely designed tissue phantoms were created, each with hard inclusions mimicking tumors. The phantoms differed in dimension, depth, and rigidity, and each of them had three inclusions. The tissue phantoms were made of a silicone blend that exhibited a Young’s modulus of approximately 5 kPa, whereas the inclusions consisted of a more rigid silicone material.

### 3.1. Size Phantom

**Figure 7:**
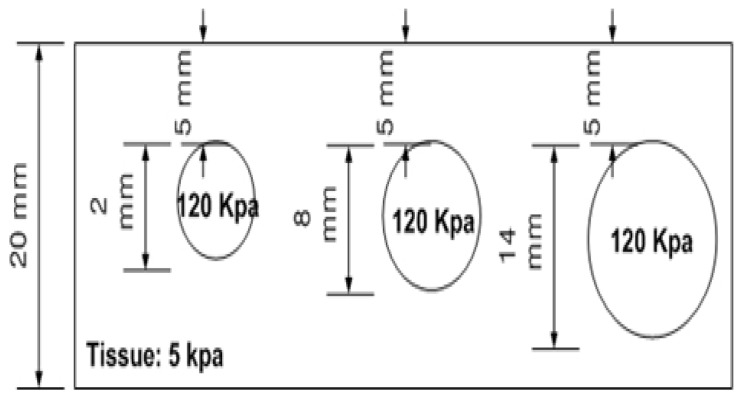
Schematic of the size phantom comprising three spherical inclusions with diameters of 2 mm, 8 mm, and 14 mm, embedded 5 mm below the surface. Each inclusion has a Young’s modulus of 120 kPa.

**Figure 8:**
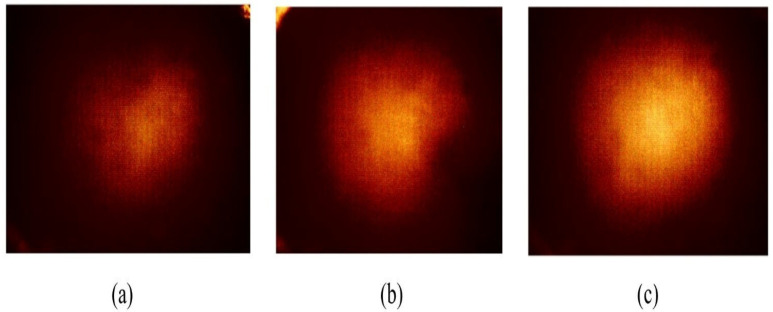
Tactile-sensation images depicting three inclusions within the size phantom. Inclusion sized at (**a**) 2 mm, (**b**) 8 mm, and (**c**) 14 mm in diameter. Images are shown within a 60° field of view with an angular scale bar (10°); pixel intensity is normalized and reported in arbitrary units (a.u.).

Three spherical inclusions of diameters of 2, 8, and 14 mm, were embedded 5 mm below the surface, each characterized by a Young’s modulus of 120 kPa. Figure 7 shows a schematic representation of the phantom size. Five tactile-sensation images were captured per inclusion and their relative diameters were calculated and averaged. Sample tactile-sensation images representing inclusions—normalized to enhance visual clarity—are shown in Figure 8.

**Figure 9:**
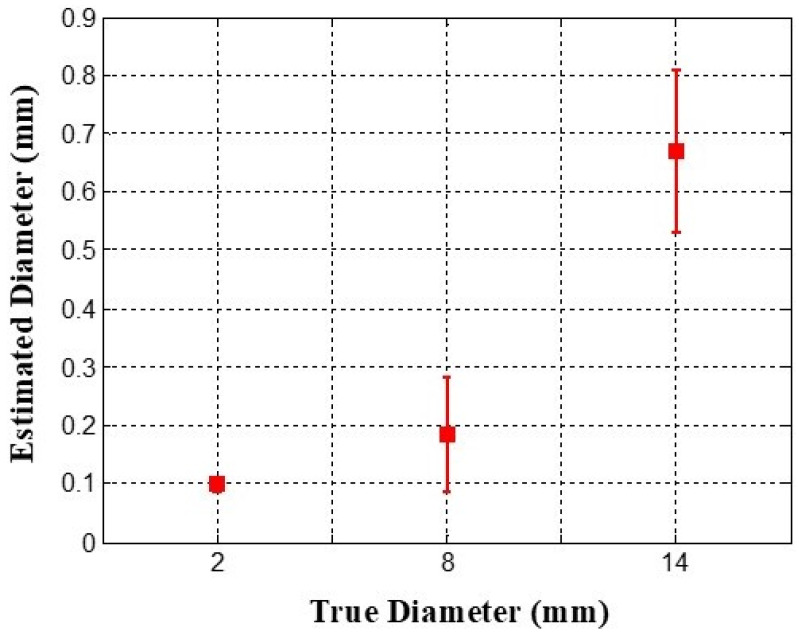
Error bar chart of estimated relative diameters of each inclusion. Data are presented as mean values with standard deviation across repeated measurements.

The diameter estimation results depicted in Figure 9 reveal that the largest inclusion, with an actual diameter of 14 mm, demonstrated the highest mean estimated diameter of 0.6697 mm, along with the greatest variability, as shown by a standard deviation of 0.2416 mm. Conversely, the smallest inclusion, measuring 2 mm in diameter, had the lowest mean estimated diameter of 0.0998 mm and the smallest variability, represented by a standard deviation of 0.0197 mm. Although the actual diameter ratio of the inclusions is approximately 1:4:7, the estimated relative diameter ratio is approximately 1:1.85:6.71.

### 3.2. Depth Phantom

**Figure 10:**
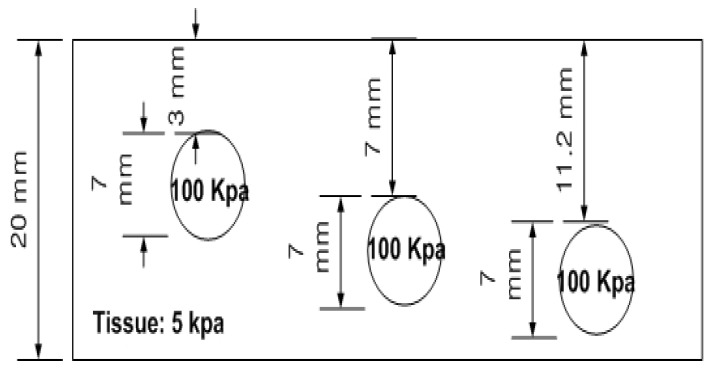
Schematic of the depth phantom comprising three spherical inclusions with a fixed diameter of 7 mm, embedded at depths of 3 mm, 7 mm, and 11.2 mm beneath the surface. Each inclusion has a Young’s modulus of 100 kPa.

**Figure 11:**
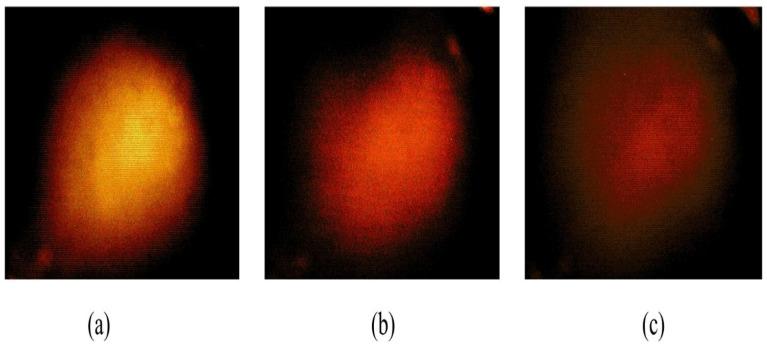
Tactile sensation images depicting three inclusions within the depth phantom: Inclusion embedded at a depth of (**a**) 3 mm, (**b**) 7 mm, and (**c**) 11.2 mm. Images are shown within a 60° field of view with an angular scale bar (10°); pixel intensity is normalized and reported in arbitrary units (a.u.).

The depth phantom consisted of three inclusions, each having a diameter of 7 mm, positioned at depths of 3, 7, and 11.2 mm, respectively. Each inclusion had a Young’s modulus of 100 kPa. A schematic representation of the depth phantom is shown in Figure 10. As with the size phantom, five tactile-sensation images were captured per inclusion, and their relative depths were calculated and averaged. Tactile-sensation images showing inclusions at varying depths are presented in Figure 11.

**Figure 12:**
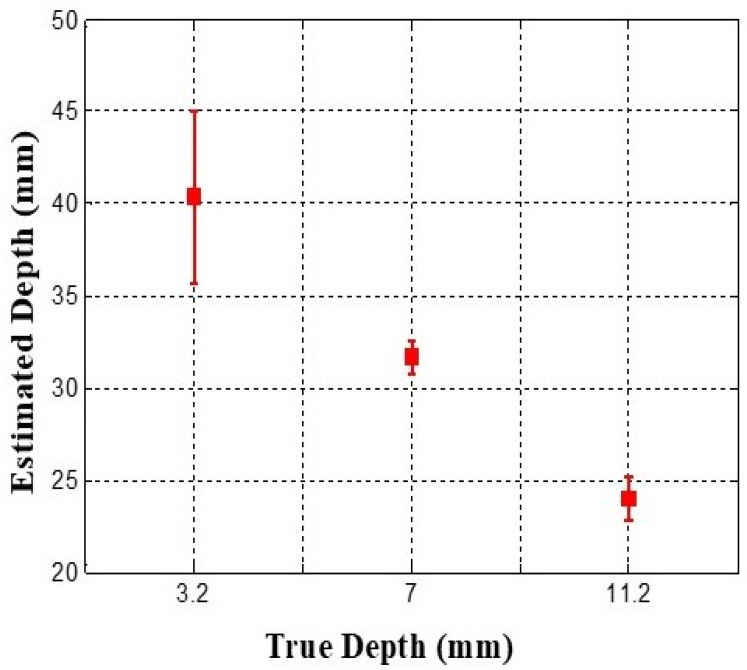
Error bar chart of estimated relative depth of each inclusion. Data are presented as mean values with standard deviation across repeated measurements.

Figure 12 illustrates the depth-estimation results, showing that the inclusion positioned at a depth of 3 mm demonstrated the highest mean estimated depth, measured at 40.33 mm, with a standard deviation of 8.08 mm. The inclusion placed at 11.2 mm depth showed the lowest mean estimated depth of 24 mm, whereas the inclusion at 7 mm depth exhibited the least variability, indicated by a standard deviation of 1.52 mm. The actual depth ratio of the inclusions was determined to be approximately 1:2.3:3.7, the estimated relative depth ratio was calculated as approximately 1:0.79:0.59. This apparent inversion between true and estimated depths is expected, as depth is inferred from surface deformation–induced scattering intensity, which decreases as the inclusion is positioned deeper beneath the surface due to reduced force transmission and waveguide deformation. As a result, the estimated metric is inversely proportional to the true inclusion depth.

### 3.3. Stiffness Phantom

**Figure 13:**
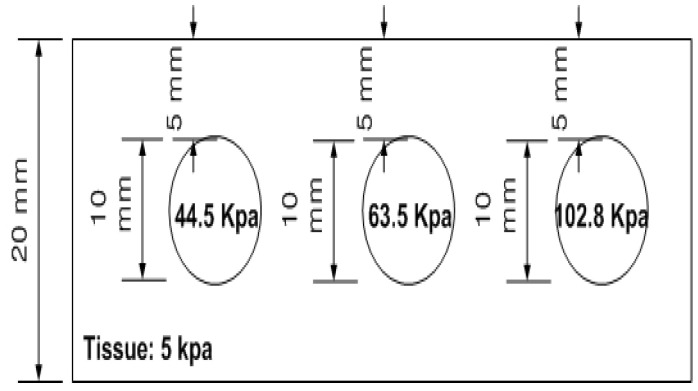
Schematic of the stiffness phantom comprising three spherical inclusions with Young’s moduli of 44.5, 63.5, and 102.8 kPa, each 10 mm in diameter and embedded 5 mm beneath the surface.

**Figure 14:**
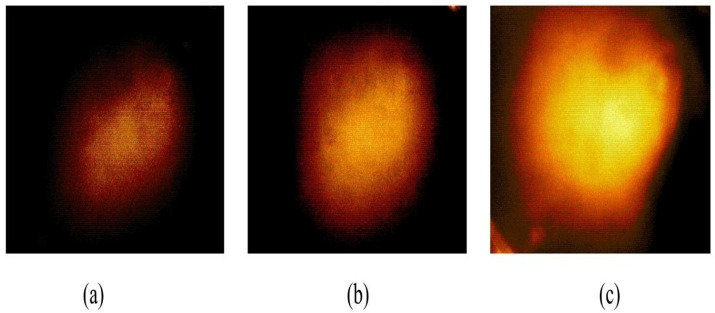
Tactile-sensation images depicting three inclusions in the stiffness phantom: Inclusion exhibiting a Young’s modulus of (**a**) 44.5 kPa, (**b**) 63.5 kPa, and (**c**) 102.8 kPa. Images are shown within a 60° field of view with an angular scale bar (10°); pixel intensity is normalized and reported in arbitrary units (a.u.).

The stiffness phantom features three inclusions, each characterized by distinct Young’s moduli: 44.5, 63.5, and 102.8 kPa. All inclusions had a diameter of 10 mm and were located 5 mm beneath the surface of the phantom. A schematic of the stiffness phantom is shown in Figure 13. Ten tactile-sensation images were acquired for each inclusion at various compression ratios to estimate and average the relative Young’s modulus. Sample tactile-sensation images representing the inclusions are shown in Figure 14.

**Figure 15:**
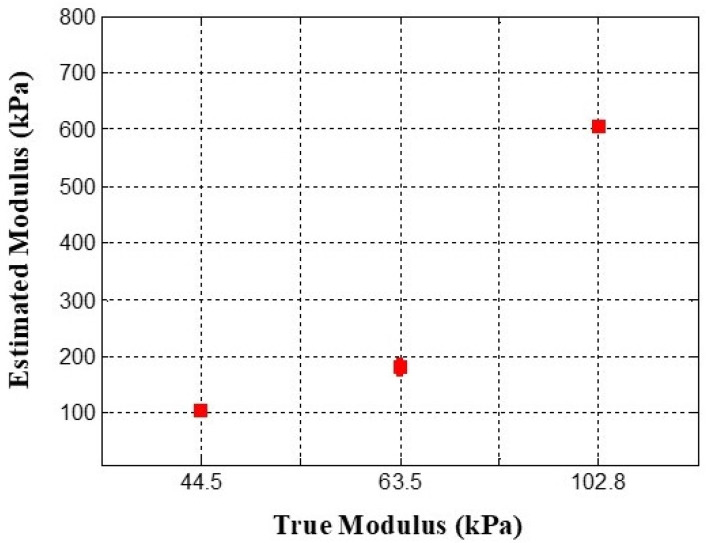
Error bar chart of relative Young’s modulus for each inclusion. Data are presented as mean values with standard deviation across repeated measurements.

The results of the Young’s modulus estimation are shown in Figure 15. The inclusion with a Young’s modulus of 102.8 kPa demonstrated the highest mean estimated modulus at 605.2 kPa, accompanied by the smallest standard deviation, reflecting a high degree of consistency in the measurements. Conversely, the inclusion with a Young’s modulus of 63.5 kPa showed the most significant variability, as illustrated in the error bar chart. The inclusion with a Young’s modulus of 44.5 kPa yielded a mean estimated modulus of 105.9 kPa, with a standard deviation of 6.66 kPa. Although the ratio of the actual Young’s moduli of the inclusions was approximately 1:1.43:2.31, the estimated ratio was approximately 1:1.71:5.71.

## 4. Discussion

This study presents the Tactile-Sensation Imaging System (TSIS), a novel optical tactile sensing approach developed to detect and characterize inclusions embedded in soft tissue. By leveraging deformation-induced optical responses, TSIS enables the extraction of relative information related to inclusion size, depth, and mechanical stiffness. The proposed approach establishes a foundation for tactile-based characterization of soft-tissue abnormalities using a compact, radiation-free sensing modality.

To achieve controlled and systematic evaluation at this early stage of development, experimental validation was conducted using tissue-mimicking phantoms. A multilayer optical waveguide was fabricated to approximate the layered structure of a human finger and total internal reflection was exploited to acquire high-resolution tactile images. The use of synthetic phantoms enabled controlled and repeatable manipulation of key parameters, including inclusion size, depth, and stiffness, while minimizing confounding biological variability. Such control is not feasible in ex vivo or in vivo tissue due to inherent heterogeneity, vascularization, and ethical constraints. Accordingly, the present results demonstrate foundational technical feasibility and relative sensitivity trends rather than clinically accurate quantitative estimation.

Several simplifying assumptions were intentionally adopted to support this proof-of-concept investigation. Depth estimation was derived from a first-order, image-based indicator under homogeneous phantom conditions rather than explicit modeling of force transmission and deformation gradients in layered tissue. Spherical inclusions were employed to provide a standardized and reproducible geometry for sensitivity and calibration assessment, consistent with established practice in phantom-based elastography and tactile imaging studies [24,25,26]. In addition, Young’s modulus estimation relied on a simplified mechanical approximation appropriate for controlled phantom experiments. While these methodological choices enable reproducible system characterization and trend analysis, they do not capture the full biomechanical complexity of biological tissue and therefore limit translational applicability.

The current implementation is subject to several limitations that influence measurement accuracy and generalizability. Nonuniform surface contact and operator-dependent pressure application may introduce variability in waveguide deformation and light scattering, particularly for softer inclusions. Depth-dependent sensitivity can lead to underestimation of stiffness for deeper inclusions due to reduced force transmission. Optical and mechanical calibration factors, including light intensity fluctuations, CMOS sensor alignment, waveguide material nonuniformity, and temperature-dependent properties of PDMS, may introduce additional sources of bias. Furthermore, tissue-mimicking phantoms do not replicate the heterogeneity, anisotropy, viscoelasticity, or nonlinear mechanical behavior of real breast tissue. Owing to the limited sample size and exploratory scope of this study, formal inferential statistics, hypothesis-driven comparisons, reliability metrics (such as test–retest reliability and intraclass correlation), confidence interval estimation, power analysis, and rigorous error propagation were not performed. As a result, the reported estimations should be interpreted as relative, phantom-calibrated indicators rather than definitive quantitative measurements or evidence of diagnostic equivalence or superiority.

Future work will focus on addressing these limitations through both methodological and experimental advancements. Improved mechanical and optical modeling, enhanced calibration strategies, and expanded phantom validation will be pursued to refine quantitative estimation. Moreover, validation against standard mechanical testing systems and extension to viscoelastic and rate-dependent models will be conducted to improve physiological relevance. In particular, testing irregular and heterogeneous inclusion geometries will be an essential next step to assess TSIS performance under more realistic tumor morphologies. Pressure-controlled actuation, depth-aware correction approaches, and machine learning-based compensation models will be explored to improve robustness, repeatability, and depth sensitivity. Rigorous statistical analysis, including confidence interval estimation, systematic bias assessment, effect size quantification, and reliability evaluation, will be incorporated using larger and more diverse datasets. Critically, validation using ex vivo and in vivo studies will be necessary to assess TSIS performance under physiologically realistic conditions that capture the heterogeneity, vascularization, and biomechanical complexity of real breast tissue. Such studies will also enable evaluation of the effects of age-related tissue changes, breast density, hormonal status, and layered anatomical variability on tactile imaging outcomes.

From a clinical perspective, TSIS is not intended to replace established breast imaging modalities such as mammography or ultrasound, but rather to complement them by providing quantitative tactile-stiffness information through surface-deformation sensing. Unlike mammography, TSIS operates without ionizing radiation and employs a compact, portable hardware con-figuration, making it potentially suitable for repeated assessments or point-of-care use. Compared with ultrasound, which relies on acoustic wave propagation and is sensitive to operator expertise, TSIS offers a direct, tactile-based measure of mechanical contrast through optical sensing. However, TSIS is inherently limited by its surface-coupled operation, resulting in reduced sensitivity to deeply located lesions, and measurement out-comes may be influenced by surface contact conditions and applied pressure. These characteristics position TSIS as a potential adjunct or preliminary assessment tool, particularly in settings where conventional imaging is unavailable, inconclusive, or impractical, with definitive diagnosis provided by established clinical modalities. Notably, the results of this study establish a technical foundation for TSIS and outline the necessary steps required to advance the system toward robust quantitative performance and meaningful clinical translation.

## Figures and Tables

**Table 1:** TSIS hardware configuration and specifications.

Component	Material/Type	Key Specifications	Function/Role
PDMS layer 1	PDMS	Hardest layer; thickness: 2 mm	Surface deformation sensing and initial force transmission
PDMS layer 2	PDMS	Intermediate hardness; thickness: 3 mm	Mechanical transition layer for load distribution
PDMS layer 3	PDMS	Softest layer; thickness: 5 mm	Enhanced deformation response to subsurface inclusions
Optical waveguide	Multilayer PDMS	Three-layer structure with graded hardness	Guides light under TIR and converts deformation into optical scattering
Camera	Monochrome cooled CMOS	Pixel size: 4.65 μm × 4.65 μm; Resolution: 1392 (H) × 1042 (V); Field of view: 60°	Captures deformation-induced scattering patterns
Protective glass	Borosilicate glass	Thermally resistant; optically transparent	Protects camera and preserves optical resolution
LED light sources	Micro-LEDs	Diameter: 3 mm; Quantity: 4; Peripheral placement	Provides illumination for total internal reflection (TIR)
Illumination configuration	Peripheral TIR illumination	Calibrated incidence angles	Ensures stable TIR conditions within the waveguide

## Data Availability

Data is contained within the article.

## Supplementary Materials

The following supporting information can be downloaded at: https://www.mdpi.com/article/10.3390/bios16020102/s1, Figure S1: Configuration of the multi-layer optical waveguide.
