# Supplementary material for: Tactile-Sensation Imaging System for Assessing Material Inclusions in Breast Tumor Detection
Source: Biosensors (Basel). 2026 Feb 4;16(2):102. doi: 10.3390/bios16020102 (PMC12938468; doi:10.3390/bios16020102)
Supplement: Supplementary file 1 [file biosensors-16-00102-s001.zip › biosensors-4075554-supplementary.pdf]

Supplemental Materials

Tactile-sensation Imaging System for Assessing Material Inclusions in Breast Tumor Detection

Analytical Solution of the Imaging Principles

This section presents the detailed analytical formulation underlying the optical and mechanical behavior of the proposed Tactile-Sensation Imaging System (TSIS). The analytical model is included to provide theoretical support for the experimental observations reported in the main manuscript and to clarify the assumptions used in interpreting deformation-induced optical responses.

We first analyzed the imaging concept based on wave-optics. Figure S1 shows the configuration of the multi-layer optical waveguide used in this system; it comprises three layers of PDMS and one layer of borosilicate glass as listed here.

1) Layer 1: PDMS, refractive value n1 and height h1,
2) Layer 2: PDMS, refractive value n2 and height h2,
3) Layer 3: PDMS, refractive value n3 and height h3,
4) Layer 4: Borosilicate-glass panel, refractive value n4 and height h4.

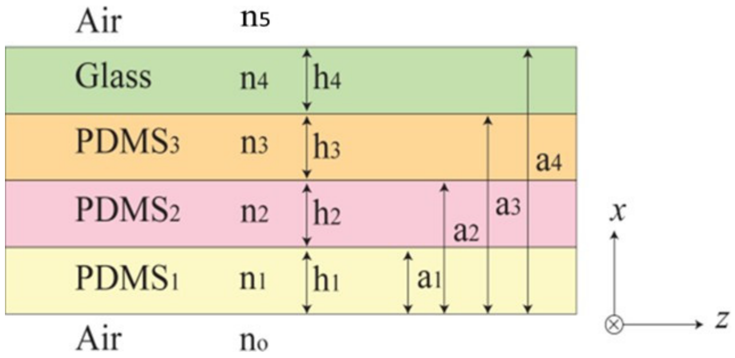

Figure S1. Configuration of the multi-layer optical waveguide.

The refractive index of the surrounding medium (air) is denoted as n_0, with $n_0 = n_5 = 1$. The optical waveguide consists of multiple layers arranged in ascending order of refractive index, such that $n_1 > n_2 > n_3 > n_4 > n_0$. Light travels along the z-axis, whereas the waveguide layers are oriented along the x-axis. For simplicity, we consider an infinite extent along the y-plane. The analysis outlines how light propagates through an optical waveguide, using Maxwell's wave equations.

$$\nabla^2 \boldsymbol{E}\,(x, y, z, t) - [n^2/c^2]\,\partial^2\,\mathbf{E}\,(x, y, z, t)\,/\partial t^2 = 0, \quad \text{(S1)}$$

where $\mathbf{E}\,(x, y, z, t)$ represents the electric field, n the refractive index of the material, and c the speed of light in a vacuum. An analogous equation governs the magnetic field $\mathbf{H}\,(x, y, z, t)$ follows:

$$\nabla^2 \boldsymbol{H}\,(x, y, z, t) - [n^2/c^2]\,\partial^2 \boldsymbol{H}\,(x, y, z, t)\,/\partial t^2 = 0. \quad \text{(S2)}$$

Because the components of electric and magnetic fields are mutually dependent, we focused on the electric field. The solution to equation (S1) can be represented for monochromatic waves at frequency ω as follows:

$$\boldsymbol{E}\,(x, y, z, t) = \boldsymbol{E}\,(x, y, z)\; exp(-i\omega t). \tag{S3}$$

Substituting this into (S1), we obtain the spatial configuration of the electric field **E** *(x, y, z)* as follows:

$$\frac{\partial^2 \boldsymbol{E}}{\partial x^2} + \frac{\partial^2 \boldsymbol{E}}{\partial y^2} + \frac{\partial^2 \boldsymbol{E}}{\partial z^2} + k_0^2 n^2 \boldsymbol{E} = 0 \tag{S4}$$

where $k_0 = \omega / c$ represents the wave vector in a vacuum. The waveguide is considered to be uniform along the z-direction, and a solution as represented by a plane wave is obtained as follows:

$$\boldsymbol{E}\,(x, y, z) = \boldsymbol{E}\,(x, y)\; exp(i\beta z), \tag{S5}$$

where β denotes the propagation constant. As the wave field is constant along the y-axis, the field distribution predominantly relies on the x-coordinate.

$$\boldsymbol{E}\,(x, y) = \boldsymbol{E}\,(x). \tag{S6}$$

The transverse y-component of the electric field was considered next, assuming the following:

$$\boldsymbol{E}\,(x) = e(x)\, j, \tag{S7}$$

where j represents the unit vector in the y direction. By substituting equation (7) into equation (4), we derive the following ordinary differential equation:

$$d^2e(x)/dx^2 + [k_0^2 n^2 - \beta^2]e(x) = 0 \tag{S8}$$

This equation applies within each layer of the optical waveguide and the surrounding air. The overall solution for the electric-field distribution in each area is

$$d^2e(x)/dx^2 + [k_0^2 n_0^2 - \beta^2]e(x) = 0, if\ x < 0 \tag{S9}$$

$$d^2e(x)/dx^2 + [k_1^2 n_1^2 - \beta^2]e(x) = 0, if\ 0 < x < a_1 \tag{S10}$$

$$d^2e(x)/dx^2 + [k_2^2 n_2^2 - \beta^2]e(x) = 0, if\ a_1 < x < a_2 \tag{S11}$$

$$d^2e(x)/dx^2 + [k_3^2 n_3^2 - \beta^2]e(x) = 0, if\ a_2 < x < a_3 \tag{S12}$$

$$d^2e(x)/dx^2 + [k_4^2 n_4^2 - \beta^2]e(x) = 0, if\ a_3 < x < a_4 \tag{S13}$$

$$d^2e(x)/dx^2 + [k_5^2 n_5^2 - \beta^2]e(x) = 0, if\ x > a_4 \tag{S14}$$

Owing to our main focus on guided modes, the solution had to decay exponentially in regions outside the waveguide while oscillating sinusoidally inside the waveguide. The general solutions for each region are as follows:

$$e = e_0\, exp[\kappa_0 x], if\ x < 0, \tag{S15}$$

$$e = e_1\, cos[\kappa_1 x + \varphi_1], if\ 0 < x < a_1, \tag{S16}$$

$$e = e_2\, cos[\kappa_2 x + \varphi_2], if\ a_1 < x < a_2, \tag{S17}$$

$$e = e_3\, cos[\kappa_3 x + \varphi_3],\, if\ a_2 < x < a_3, \tag{S18}$$

$$e = e_4\, cos[\kappa_4 x + \varphi_4],\, if\ a_3 < x < a_4, \tag{S19}$$

$$and\ e = e_5\, exp[\kappa_5(h1 + h2 + h3 + h4 - x)],\, if\ x > a_4. \tag{S20}$$

The solutions must satisfy the boundary conditions and match the fields and their derivatives at each interface between the layers. Substituting these solutions into equation (S9)–(S14) yields the following dispersion relationship:

$$-k_0^2 + \beta^2 = k_0^2\ if\ x < 0, \tag{S21}$$

$$k_1^2 + \beta^2 = k_0^2 n_1^2\ if\ 0 < x < a_1, \tag{S22}$$

$$k_2^2 + \beta^2 = k_0^2 n_2^2\ if\ a_1 < x < a_2, \tag{S23}$$

$$k_3^2 + \beta^2 = k_0^2 n_3^2\ if\ a_2 < x < a_3, \tag{S24}$$

$$k_4^2 + \beta^2 = k_0^2 n_4^2\ if\ a_3 < x < a_4, \tag{S25}$$

$$and\ -k_5^2 + \beta^2 = k_5^2\ if\ x > a_4. \tag{S26}$$

To fully explore the imaging principle, the boundary conditions and matching field components must be applied. To achieve this, we first obtain the magnetic field from Maxwell's equation related to the curl of E, whose structure is comparable to the electric field but is limited to only one non zero component in the z-direction:

$$\boldsymbol{H}(x, y, z, t) = \boldsymbol{k}\, h(x)\, exp\,(-i\beta z + i\omega t) \tag{S27}$$

where $\boldsymbol{k}$ is a unit vector in the z-direction. Substituting this expression into Maxwell's curl equation for $\boldsymbol{E}$ we obtain

$$curl\, E = -(1/c)\, \partial \boldsymbol{H}/\partial t. \tag{S28}$$

This results in the following general solution for the magnetic field, which reflects the parameters utilized for the electric field:

$$h(x) = -(ic/\omega)\, \kappa_0 e_0\, exp[\kappa_0 x]\ if\ x < 0, \tag{S29}$$

$$h(x) = (ic/\omega)\, \kappa_1 e_1\, sin[\kappa_1 x + \varphi_1]\ if\ 0 < x < a_1 \tag{S30}$$

$$h(x) = (ic/\omega)\, \kappa_2 e_2\, sin[\kappa_2 x + \varphi_2]\ if\ a_1 < x < a_2, \tag{S31}$$

$$h(x) = (ic/\omega)\, \kappa_3 e_3\, sin[\kappa_3 x + \varphi_2]\ if\ a_2 < x < a_3, \tag{S32}$$

$$h(x) = (ic/\omega)\, \kappa_4 e_4\, sin[\kappa_4 x + \varphi_4]\ if\ a_3 < x < a_4, \tag{S33}$$

$$and\ h(x) = (ic/\omega)\, \kappa_5 e_5\, exp[\kappa_5(a_4 - x)]\ if\ x > a_4. \tag{S34}$$

To ensure continuity of the field components across boundaries, the impedance h/e must remain continuous at each boundary for $x = 0$, $x = a_1$, $x = a_2$, $x = a_3$, $x = a_4$. From this, we obtain the following matching conditions:

$$k_0 = -k_1\, tan(\varphi_1)\ if\ x = 0 \tag{S35}$$

$$k_1\, tan(\kappa_1 a_1 + \varphi_1) = k_2\, tan(\kappa_2 a_1 + \varphi_2)\ if\ x = a_1 \tag{S36}$$

$$k_2\, tan(\kappa_2 a_2 + \varphi_2) = k_3\, tan(\kappa_3 a_2 + \varphi_3)\ if\ x = a_2 \tag{S37}$$

$$k_3\, tan(\kappa_3 a_3 + \varphi_3) = k_4\, tan(\kappa_4 a_3 + \varphi_4)\ if\ x = a_3 \tag{S38}$$

$$and\ k_4\, tan(\kappa_4 a_4 + \varphi_4) = k_5\ if\ x = a_4 \tag{S39}$$

The spectrum of the guided modes in an optical waveguide is defined by these equations, which can be consolidated into a single transcendental equation involving one variable.

$$k_2\, sin(\kappa_2 a_2 + \varphi_2)\, cos(\kappa_3 a_2 + \varphi_3) = k_3\, sin(\kappa_3 a_2 + \varphi_3)\, cos(\kappa_2 a_2 + \varphi_2) \tag{S40}$$

where the subsequent substitutions need to be applied for each boundary condition:

$$\varphi_1 = -arctan(k_0/k_1) \tag{S41}$$

$$\varphi_2 = \arctan\left[(k_1/k_2)\, tan(\kappa_1 a_1 + \varphi_1)\right] - \kappa_2 a_1 \tag{S42}$$

$$\varphi_3 = arctan\left[(k_4/k_3)\, tan(\kappa_4 a_3 + \varphi_4)\right] - \kappa_3 a_3 \tag{S43}$$

$$and\ \varphi_4 = arctan(k_0/k_4) - \kappa_4 a_4 \tag{S44}$$

Here, the transverse wave vectors k_i are calculated as follows:

$$k_0 = \sqrt{\beta^2 - k^2{}_0} \tag{S45}$$

$$k_1 = \sqrt{k_0^2 n_1^2 - \beta^2} \tag{S46}$$

$$k_2 = \sqrt{k_0^2 n_2^2 - \beta^2} \tag{S47}$$

$$k_3 = \sqrt{k_0^2 n_3^2 - \beta^2} \tag{S48}$$

$$k_4 = \sqrt{k_0^2 n_4^2 - \beta^2} \tag{S49}$$

$$k_5 = \sqrt{\beta^2 - k^2{}_5} \tag{S50}$$

The boundary conditions also allow for determining the electric-field amplitudes, e_i within each layer:

$$e_1 = e_0/cos(\varphi_1)\ if\ x = 0 \tag{S51}$$

$$e_2 = e_1 cos(\kappa_1 a_1 + \varphi_1)/cos(\kappa_2 a_1 + \varphi_2) \ if \ x = a_1 \quad \text{(S52)}$$

$$e_3 = e_2 cos(\kappa_2 a_2 + \varphi_2)/cos(\kappa_3 a_2 + \varphi_3) \ if \ x = a_2 \quad \text{(S53)}$$

$$e_4 = e_3 cos(\kappa_3 a_3 + \varphi_3)/cos(\kappa_4 a_3 + \varphi_4) \ if \ x = a_3 \quad \text{(S54)}$$

$$e_5 = e_4 cos(\kappa_4 a_4 + \varphi_4) \ if \ x = a_4 \quad \text{(S55)}$$

The amplitude, e_0 is set by the intensity of the light introduced into the optical waveguide. After substituting these values into equation (S40), the only unknown left is the propagation constant β, whose solution reveals the complete spectrum of light propagation within the waveguide.
